# Assessing the Skin Sensitizing Potential of Constituents and Leachables from Wearable Devices Using GARDskin and Associated Applications

**DOI:** 10.3390/toxics14100928

**Published:** 2026-10-20

**Authors:** Ravi Persaud, Andy Forreryd, Robin Gradin, Emma Anders, Johan Andersson, Per Alftrén, Henrik Johansson, Nakul Ruparel

**Affiliations:** 1 Apple, Inc., Cupertino, CA 95014, USA; 2 SenzaGen AB, SE-22481 Lund, Sweden; robin.gradin@senzagen.com (R.G.); emma.anders@senzagen.com (E.A.); johan.andersson@senzagen.com (J.A.); per.alftren@senzagen.com (P.A.); henrik.johansson@senzagen.com (H.J.)

**Keywords:** wearable devices, skin sensitization, GARD, quantitative potency assessment

## Abstract

Pre-clinical toxicological evaluation is an essential component of ensuring the safety and compatibility of wearable devices with biological systems. Along with the local endpoints of cytotoxicity and irritation, skin sensitization is a core toxicological endpoint. Testing wearable devices, which are often solid and composed of multiple components, presents new challenges for New Approach Methods. In the current proof-of-concept study, we propose a strategy for conducting risk assessment of the individual components and the leachables from wearable devices. The GARDskin Dose-Response assay (OECD TGP 4.106) was used to predict the skin sensitization potency of three acrylate monomers, two acrylamide monomers, and one reactive dye. The GARDskin Medical Device (ISO 10993-10:2021, Annex C) was used to classify MED-2000 extracts spiked with isobornyl acrylate. All chemicals were classified as skin sensitizers. The quantitative potency predictions correlated well with available data from defined approaches and produced near-identical potency rankings. Available human and animal reference data were too sparse and heterogenous to substantiate agreement in potency. Finally, the GARDskin Medical Device assay correctly classified the spiked extract as skin sensitizing. In conclusion, the GARDskin assay and associated applications are well adapted to address the testing requirements for wearable devices.

## 1. Introduction

A wearable device, or wearable in short, may be defined as a body-worn electronic technology equipped with sensors, data processing capability, and connectivity functions, designed to continuously collect, analyze, and communicate information about the wearer’s physiological state, behavior, or surrounding environment [1,2]. Depending on the specific intended purpose, wearables may or may not be classified as medical devices. Once a wearable is classified as a medical device, products fall under the European Union’s Medical Device Regulation (2017/745) [3] or corresponding regulations by the U.S. Food and Drug Administration (FDA), requiring biocompatibility testing according to the International Organization for Standardization (ISO) 10993 series of standards, the framework of which is described in ISO 10993-1 [4]. Furthermore, when a classification as a medical device is inappropriate, products are subject to general consumer safety and product compliance requirements, including chemical safety regulations such as Restriction of Hazardous Substances (RoHS, Directive 2011/65/EU) [5] and Registration, Evaluation, Authorisation and Restriction of Chemicals (REACH, Regulation (EC) No 1907/2006) [6].

A key biological endpoint (i.e., adverse effect) to be assessed under REACH and ISO 10993 for all medical devices, regardless of intended use, is skin sensitization. A skin sensitization hazard is defined as an agent that can induce sensitization, i.e., a type IV hypersensitivity reaction [7], in exposed individuals. The immunological mechanisms underlying skin sensitization are currently well understood [8,9,10], and testing strategies to assess the skin sensitization potential of chemicals have evolved rapidly over the last decade. Following the publication of the Organisation for Economic Co-operation and Development (OECD) Adverse Outcome Pathway (AOP) [11], a series of in vitro test methods have been validated and adopted into the OECD Test Guidelines (TG) 442C-E [12,13,14]. While the use of such techniques has been widely adopted, e.g., in the cosmetic and chemical sectors, the medical device sector remains reliant mainly on traditional in vivo methods, such as guinea pig test methods [15] or the murine Local Lymph Node Assay (LLNA) [16].

To accelerate the adoption of novel in vitro methods in the industry, as well as promote the regulatory acceptance of such New Approach Methods (NAMs), the ISO TC 194 recently published a Technical Specification (TS), ISO TS 11796 [17], which describes the process proposed to qualify already validated OECD TG methods for adoption into the nominal parts of ISO 10993-10 [18]. Here, a method is proposed to be qualified for regulatory use following a feasibility study, a pre-validation study, and a validation study.

In the context of risk assessment of wearables for skin sensitization, the Genomic Allergen Rapid Detection platform (GARD), and the associated GARDskin method [14], is a particularly attractive tool. Firstly, it has demonstrated a high predictive performance in a series of (pre-)validation studies [14,19,20] and a wide applicability domain of relevance to the wearable segment, including maintained predictive accuracy in so-called difficult-to-test chemicals and complex formulations [21,22] and metals [23]. Secondly, the method has been subject to adaptations, i.e., protocol extensions, referred to as GARDskin Medical Device (MD) and GARDskin Dose-Response (DR), which facilitate testing of solid materials [24] and the quantitative assessment of sensitizing potency [25,26], respectively. Indeed, the capabilities of these methods were recently demonstrated, with the GARDskin MD method having completed a feasibility study [24] and with pre-validation studies ongoing. As such, it constitutes, as of to date, the most advanced method in terms of progression along the process defined by ISO TS 11796, moving forward with an interlaboratory validation study as per the available guidance. Similarly, GARDskin DR has been utilized in a series of potency (sub-)classifications applications, ranging in resolution from predicting LLNA EC3 categories and UN GHS CLP subcategories to estimating a Point of Departure (PoD) on a continuous scale [26,27,28], features which otherwise typically require integrating results from several data sources [29,30,31].

In terms of chemicals released from medical devices and wearables, the literature often lacks sufficient information to establish thresholds for the induction of skin sensitization. Evaluating sensitization potency and determining an appropriate PoD are critical for assessing the safety of both new and existing chemicals to which workers and consumers may be exposed. In recent years, numerous case reports have highlighted concerns regarding materials used in the manufacture of wearable products that have been associated with allergic contact dermatitis (ACD).

The chemical Solvent Orange 60 (SO60) is a perinone-type dye often used in plastic materials such as spectacle frames, goggles, and gloves, and it has been shown to cause contact allergy [32,33,34,35,36,37,38,39,40,41,42]. Isobornyl acrylate (IBOA) and N, N-dimethylacrylamide (NNDMA) are monomers used in medical devices such as glucose sensors or insulin pumps and have been reported to cause contact allergic reactions [41,43,44,45,46,47,48,49,50,51,52,53,54]. These adhesive monomers apparently leached from the plastic encasing the device and sensitized patients over prolonged exposure. Acryloylmorpholine (ACMO) was identified in an outbreak of occupational ACD involving workers exposed to adhesives in a smartphone protective case [55,56]. Similarly, ACMO and IBOA have been associated with ACD from phone protectors. ACMO is structurally related to NNDMA; however, it remains uncertain whether cross-reactions occur between them in practice. Hydroxypropyl methacrylate (HPMA) is repeatedly reported to cause allergic contact dermatitis, including among nail technicians and users of long-lasting nail polish [57]. In addition, 2,3-Epoxypropyl methacrylate is reported as a cause of occupational allergic contact dermatitis in workers handling liquid acrylates, including a case of severe bullous ACD confirmed by patch testing [58].

In the present study, GARDskin and its adaptations were explored for their utility in testing scenarios related to the safety assessment of wearables. Similar to recent work [59], a set of sector-relevant test chemicals, including the six materials specified above, was assessed for sensitizing potency using the GARDskin DR method to determine a PoD for downstream risk assessment. Furthermore, a case study was conducted on IBOA to evaluate the method’s ability to detect such chemistry in complex formulations resulting from ISO 10993-12 [60] extraction procedures.

## 2. Materials and Methods

### 2.1. GARDskin

The GARDskin method [14] is a validated NAM for the hazard assessment of skin sensitizers, adopted in OECD TG 442E. The complete set of GARDskin protocols is publicly available [61] but can be conceptually summarized as follows.

The human myeloid leukemia SenzaCell cell line (ATCC Depository PTA-123875) is used as an in vitro model of dendritic cells (DCs). As such, the method monitors the immunological activation of DCs in response to xenobiotics following test item exposure. Cells in steady-state growth are exposed to test chemicals at concentrations that yield low- to non-toxic effects, with a maximum concentration of 500 µM if molar concentrations are known or otherwise defaulted to 100 ppm. Following 24 h exposure, total mRNA is isolated from cell cultures and analyzed using NanoString nCounter instrumentation [62]. Specifically, the transcriptional levels of a set of 196 genomic biomarkers, referred to as the GARDskin Genomic Prediction Signature (GPS) [63], are quantified and fed into a prediction algorithm [64] based on a Support Vector Machine (SVM) [65]. Lastly, the output from the prediction algorithm, referred to as a Decision Value (DV), is fed into the final prediction model, which classifies any test chemical with a mean DV ≥ 0 (n = 3) as a sensitizing hazard. Consequently, any test chemical with a mean DV < 0 is classified as a non-sensitizer.

The current work is primarily based on data generated with adapted versions of the GARDskin method. These adaptations are referred to as the GARDskin DR method, which facilitates quantitative assessment of sensitizing potency, and the GARDskin Medical Device method, which enables testing of solid materials in accordance with ISO 10993 standards. Each of these adaptations is described separately in the sections below.

### 2.2. GARDskin Dose-Response

The GARDskin DR method is an extension of the conventional GARDskin method, in which test chemicals are evaluated by the GARDskin prediction algorithm in an extended range of concentrations (n = 6 concentrations, 2 replicates per concentration), to investigate the dose-response relationship between GARDskin DVs and test chemical concentration. As such, it provides a quantitative estimate of sensitizing potency, referred to as cDV_0_, which corresponds to the lowest dose required to generate a positive mean DV. The method was first proposed by Gradin et al. [25] and further refined by Gradin et al. [26]. In addition, the finalized protocol has been demonstrated to be functional across a series of applications related to different risk assessment scenarios [26,27,28].

In brief, following the establishment of a test chemical-specific input concentration, as described above, the identified concentration is defaulted as the top concentration in a titrated dose range so that additional test concentrations are also evaluated in a series of concentrations below the top concentration. By identifying the highest concentration yielding a negative mean DV and the lowest concentration yielding a positive mean DV, the cDV_0_ can be obtained from linear interpolation between the two data points. Depending on the purpose of testing and downstream risk assessment strategies, the cDV_0_ can be further converted to a Predicted Potency Value (PPV) in μg/cm^2^. As such, the PPV can be further used as a direct estimate of the No Effect Sensitization Induction Level (NESIL) and evaluated, e.g., for CLP 1A/1B subcategorization [66] by relating the obtained values to the ECOTOC classification threshold of 500 µg/cm^2^. For additional details on these procedures, the interested reader is referred to reference [26].

### 2.3. GARDskin Medical Device

The GARDskin Medical Device (MD) method is an adaptation of the conventional GARDskin method that facilitates testing of solid materials in compliance with the ISO 10993 series of standards. Specifically, the adapted method ensures compatibility with the solid material extraction procedures specified by ISO 10993-12 [60] for testing the skin sensitization hazard of medical devices, as outlined by ISO 10993-10 [18], first proposed and described in detail by Jenvert et al. [24].

In brief, solid test materials (e.g., components of wearables or medical devices) are the subject of extraction procedures specified in ISO 10993-12. Extraction procedures are performed using a polar solvent (e.g., a buffered saline solution) and a non-polar solvent (e.g., a vegetable oil), yielding two test items per test material. Similarly to the conventional GARDskin method, cells are exposed to these dual test items at steady-state growth, at low- to non-toxic concentrations and with a constant total volume of extraction vehicle. In essence, all downstream sample handling and genomic analyses are performed identically as the conventional GARDskin method, except for an adaptation of the final prediction model to account for the dual test items. If one, or both, of the polar or non-polar test items originating from the same test material is assigned a mean DV ≥ 0, the test material is classified as a sensitizing hazard. For additional details on these procedures, the interested reader is referred to [24].

### 2.4. Test Chemicals and Reagents

**Figure 1:**
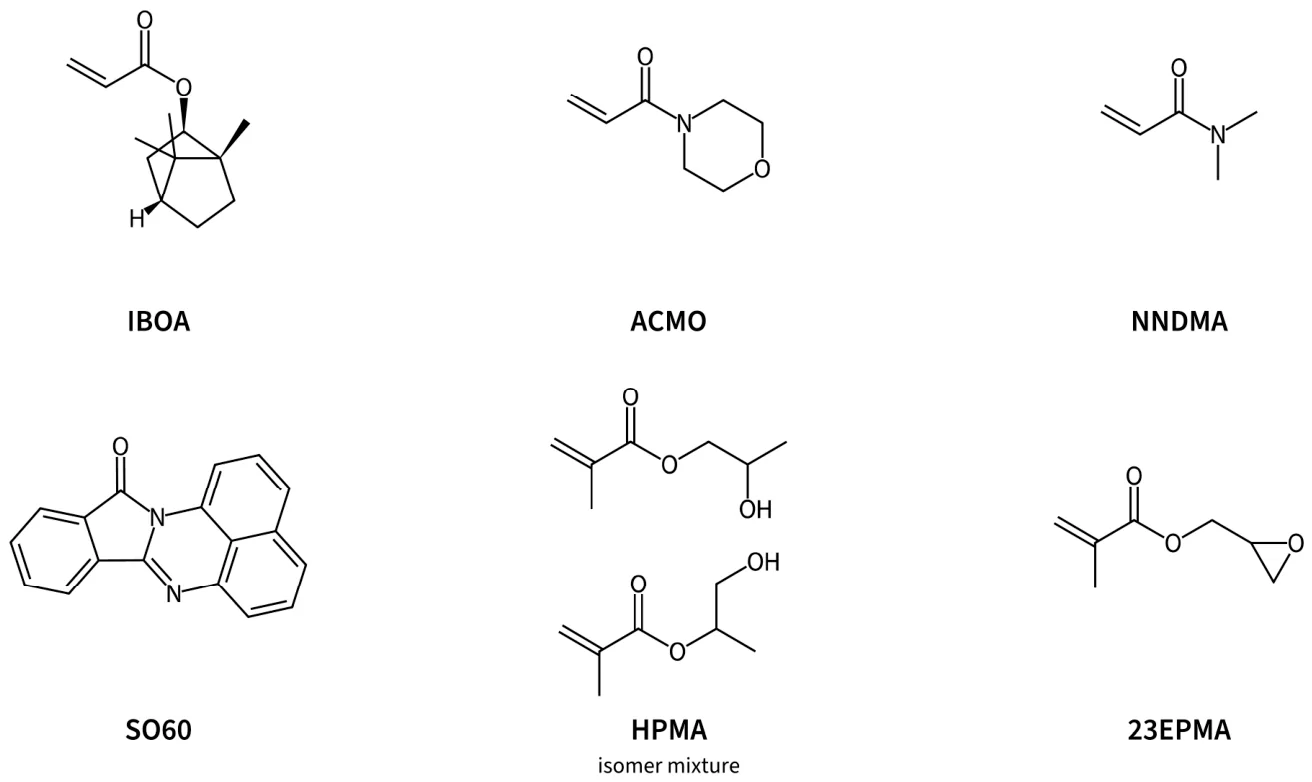
Molecular structures of the test chemicals.

The test chemicals investigated in this study were isobornyl acrylate (abr: IBOA, CAS: 5888-33-5), 4-acryloyl morpholine (abr: ACMO, CAS: 5117-12-4), N,N-dimethyl acrylamide (abr: NNDMA, CAS: 2680-03-7), solvent range 60 (abr: SO60, CAS: 6925-69-5), hydroxypropyl methacrylate (abr: HPMA, CAS: 27813-02-1), and 2,3-epoxypropyl methacrylate (abr: 23EPMA, CAS: 106-91-2), see structures in Figure 1. The chemicals were chosen to reflect a typical chemical domain of relevance for the wearables industry segment, as well as due to the availability of reference data, which were extracted from Ruparel et al. [59]. A more comprehensive discussion of the available in vivo references is available in Supplementary Material S1. All chemicals were purchased from Sigma-Aldrich (St. Louis, MO, USA).

For the GARDskin MD case study of IBOA, cured and hardened MED-2000 material (kindly donated by Dr. Kelly Coleman, Medtronic, MN, USA) was used as a proxy test material. Extraction procedures according to ISO 10993-12 were conducted using super-refined olive oil (CAS: 8001-25-0) (Croda, Snaith, UK) and saline (0.9% NaCl) solution (CAS: 7647-14-5) (G-Biosciences, St. Louis, MO, USA) supplemented with 1% PEST (Penicillin-Streptomycin solution 100×, L0022; Biowest, Nuaillé, France) as non-polar and polar extraction vehicles, respectively.

For details on additional reagents and consumables used in the methods, the interested reader is referred to published protocols [61].

### 2.5. Proxy Test Material Extractions and Extract Sample Preparations

To create a case study on detecting IBOA in complex extract samples, proxy samples were prepared according to the guidance in ISO TS11796 [17]. MED-2000, a commonly used medical-grade silicone material, was used as a reference test material and used to prepare reference extract samples using polar and non-polar extraction vehicles, respectively, according to ISO 10993-12 [60]. In short, 0.2 g of the material was incubated with rotation at 37 °C (±1 °C) for 72 h (±2 h) in 1 mL saline supplemented with 1% PEST and 1 mL super-refined olive oil. Following extraction, reference extract samples were spiked with isobornyl acrylate at a concentration corresponding to the LLNA EC3 value (1%). Spiked extracts were considered proxy samples representing a test material containing a sensitizing agent and were subjected to downstream testing in the GARDskin MD method.

### 2.6. SARA-ICE POD Predictions

The Skin Allergy Risk Assessment–Integrated Chemical Environment (SARA-ICE)-defined approach [31] was used to predict skin sensitization points of departure (PoDs) based on the ED01 endpoint, defined as the dermal dose associated with a 1% probability of inducing skin sensitization in a human predictive patch test exposure scenario. Input data were obtained from Ruparel et al., 2024 [59], comprising complete kDPRA and KeratinoSens assay results for IBOA, SO60, ACMO, and NNDMA. As noted previously, the kDPRA SO60 data showed non-linear reaction kinetics in the kDPRA assay [67,68]. Despite the rate constant being derived using standard protocols for non-linear scenarios, its value was considered of relatively lower reliability in this study. No compatible NAM-derived input data were identified for HPMA or 23EPMA. All input data are provided in the Supplementary Materials. SARA-ICE predictions were generated using the SARA-ICE Tool (https://ntp.niehs.nih.gov/sites/default/files/sara-ice/?NTPW-4081-DEPLOY; accessed on 6 May 2026), selecting the OECD TG 497 Defined Approach model version. The generated prediction report is attached as Supplementary Materials.

### 2.7. Graphical Representation of Data

Graphical representations of data were created using R, version 4.4.3 [69], with ggplot2 version 4.0.0 [70].

## 3. Results

### 3.1. GARDskin Dose-Response Classifications (Binary Hazard Assessments, UN GHS Sub-Categorizations, and NESIL Predictions)

**Figure 2:**
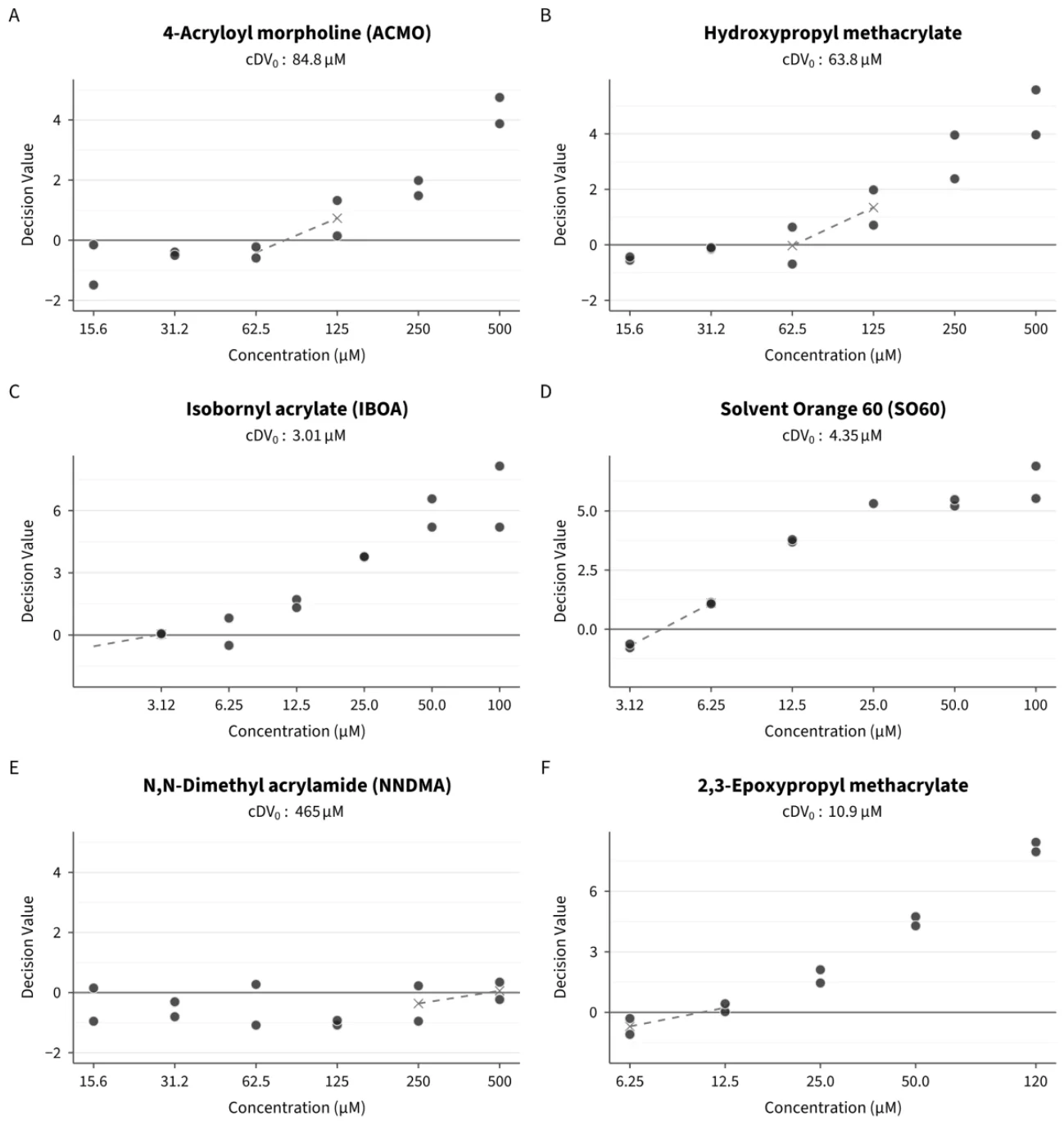
GARDskin DR results of test chemicals (**A**) 4-acryloyl morpholine (**B**) hydroxypropyl methacrylate (**C**) isobornyl acrylate (**D**) solvent orange (**E**) N-N dimethyl acrylamide (**F**) 2,3-epoxypropyl methacrylate. Dashed lines connect concentrations used to estimate cDV_0_ values using linear interpolation. Points describe observations, and crosses describe mean values used for interpolation. *X*-axis labels describe assayed concentrations.

The GARDskin DR assay is based on the validated protocols of GARDskin (OECD TG 442E) and provides binary hazard classifications and potency sub-categorizations according to the UN GHS (1A and 1B), as well as potency predictions on a continuous scale corresponding to a human NESIL value (μg/cm^2^). In the current study, six materials were evaluated using the established GARDskin DR protocol, and the resulting dose-response curves were used to determine the cDV_0_ concentration and characterize the skin sensitization potential. As evident in Figure 2, all chemicals induced a monotonic dose-dependent increase in the response value (DVs). In addition, except for the chemical IBOA, for which no negative mean DVs were observed, all test chemicals exhibited both negative and positive mean DVs within the assayed concentration interval, enabling the identification of a cDV_0_ value according to established standard protocols. For IBOA, the lack of any negative mean values indicated a strong sensitization potential. In such cases, and analogous to the protocol for the LLNA, the standard operating procedures for the GARDskin DR enabled interpolation between the first positive DV value (DV > 0) and the DV for the unstimulated control sample (concentration = 0). The resulting binary hazard classifications, UN GHS sub-categories, and predicted NESIL values are summarized in Table 1. As apparent from the table, all test chemicals were classified as skin sensitizers, but there was approximately a two-orders-of-magnitude difference in predicted skin sensitizing potency between the most potent (IBOA) and the least potent chemical (NNDMA) in the dataset.

### 3.2. Comparison of GARDskin Dose-Response Classifications to Available Reference Data

The potency predictions derived from the GARDskin DR assay were compared with published data. For most chemicals, limited reference data from animal or human studies could be identified in the public domain, as summarized in Table 1. A more detailed review of the available reference data for each test chemical is available in Supplementary Material S1.

**Figure 3:**
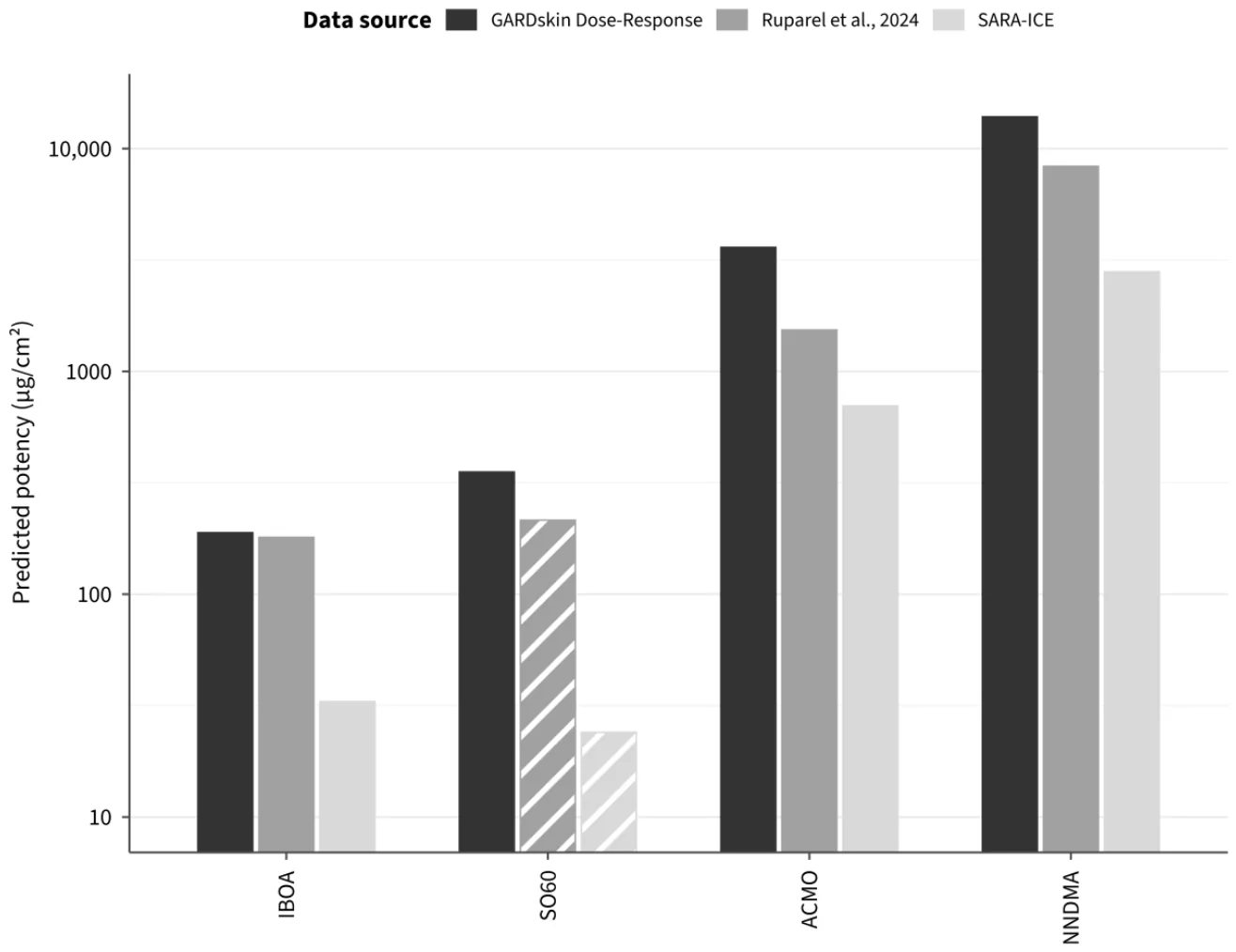
Comparison of predicted potency values for overlapping test chemicals, generated by GARDskin DR, SARA-ICE, and extracted from Ruparel et al., 2024 [59]. Striped bars describe DA predictions with relatively higher uncertainty due to the lower reliability of the kDPRA rate constant estimated from non-linear reaction kinetics.

Despite the relatively low amount of reference data available in the scientific literature, which in most cases prevented the derivation of a reliable potency estimate, published NAM data were available for a total of four test materials from a similar study [59], investigating the potential to generate quantitative potency predictions based on the readout from a combination of OECD-based assays using the regression models described in Natsch et al. [29]. In addition, SARA-ICE PoD predictions were generated using the same NAM results described in that study. Comparing the PPVs from the standalone GARDskin DR model to the potency predictions derived from the regression model based on a combination of assays, the two models provided very similar predictions, as shown in Figure 3. As can also be seen, the SARA-ICE PoDs were consistently more conservative than either the GARDskin DRor the regression model. In addition, the SARA-ICE model ranked SO60 as the strongest sensitizer, while both GARDskin DR and the regression model assigned the strongest potency to IBOA. Notably, the relative differences in potency between SO60 and IBOA were small for all models.

### 3.3. GARDskin Medical Device: Testing in Polar and Non-Polar Vehicles in Accordance with ISO 10993-12

**Figure 4:**
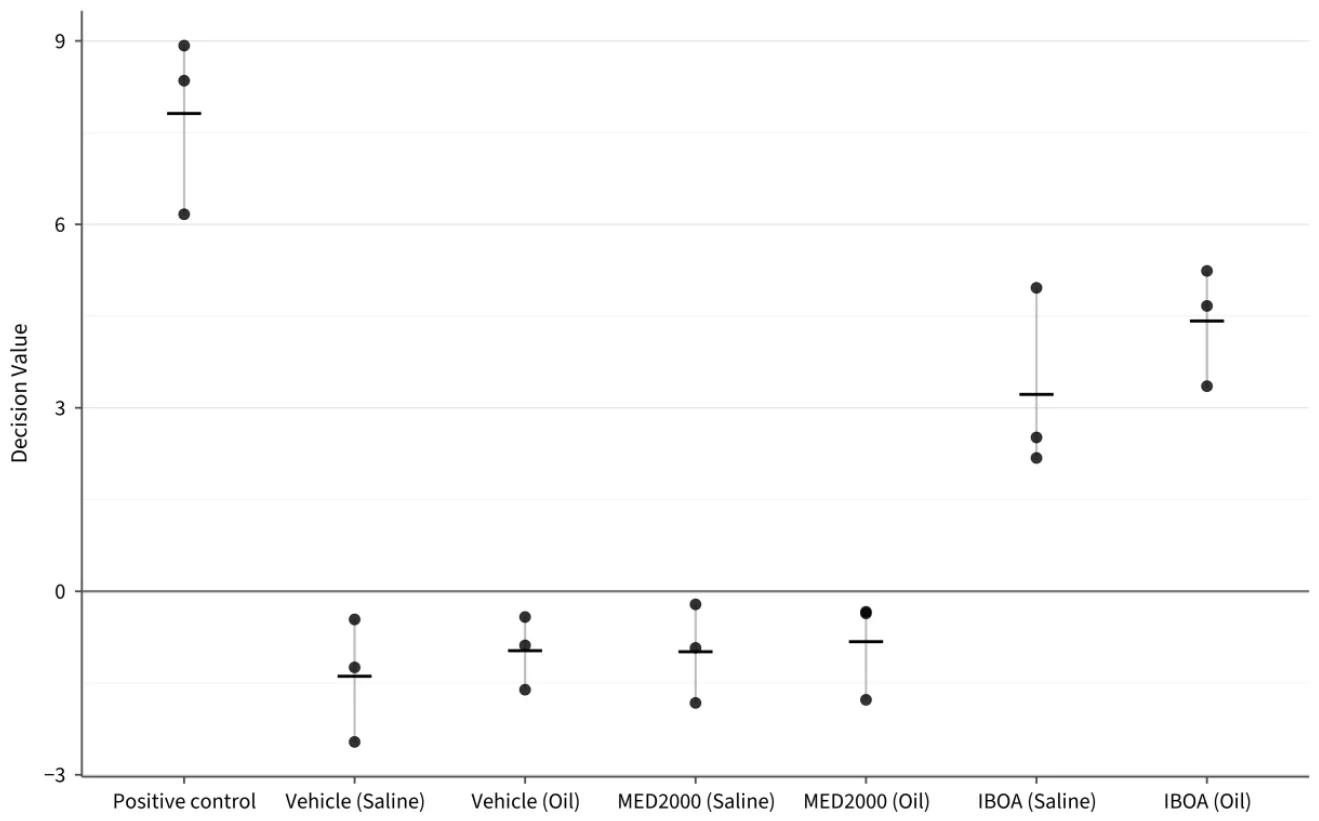
GARDskin MD results of MED-2000 extracts spiked with IBOA. The vehicle controls for saline and oil are used as negative controls.

Most wearables designed for direct or indirect contact with human skin are either solid or semi-solid. Thus, to pose a significant risk to humans, chemicals must leach from the material, become bioavailable, and be accessible to the cells of the immune system to trigger downstream events. To mimic the relevant exposure conditions of use, ISO 10993-12 provides guidance on preparing solid medical devices for testing. Although wearables are not regulated in the same way as medical devices, the method preparation can be guided by the principles established in the ISO standard. Hence, to evaluate the potential of the GARDskin method to also detect leachables from wearables in a realistic setting, a proof-of-concept study was conducted using the chemical IBOA. The chemical was spiked into polar (saline) and non-polar (olive oil) extracts of the MED-2000 material, and the extracts were evaluated using the GARDskin Medical Device approach. As illustrated in Figure 4, IBOA was clearly positive in both the polar and the non-polar extracts. In contrast, the blank (i.e., non-spiked) MED-2000 extracts were clearly negative, indicating the potential to detect skin sensitizing hazard in extracts from wearables.

## 4. Discussion

Skin sensitization is a hazardous endpoint of concern for a wide range of industrial sectors and products, including chemicals, cosmetics and fragrances, agricultural products, pharmaceuticals, and medical devices. Testing strategies for skin sensitization assessment have evolved rapidly in the last two decades, transitioning from traditional in vivo testing to the so-called NAMs, as evidenced by the growing number of OECD TGs (i.e., TG 442C-E), integrated testing strategies (ITS), and defined approaches (DA) guiding the subject. However, the wearables’ product segment may pose a particular challenge to adequately test, due to the products’ solid nature and an idiosyncratic regulatory landscape that combines definitions from the risk assessment of neat chemicals and regulations associated with medical devices.

In the herein-presented study, GARDskin and associated protocol adaptations, GARDskin DR and GARDskin MD, were evaluated for their relevance and adequacy for testing chemical compounds typically associated with the wearable product segment. Specifically, GARDskin DR was used to predict hazard and quantitative sensitizing potency for a set of six test chemicals. Similarly, GARDskin MD was used in a case study, based on spiking MED-2000 extracts with IBOA, a compound frequently used by the industry and reported as a probable cause for clinical symptoms of allergic contact dermatitis, to demonstrate its functionality in terms of detecting hazards in complex formulations associated with testing according to the ISO 10993 series of standards.

Based on the initial hazard assessments, all six test chemicals were classified as skin sensitizers. Based on available clinical human evidence, all six test chemicals are considered sensitizing hazards. Thus, GARDskin was shown to provide experimental results in line with the clinical expectation. Indeed, the results are noteworthy, as traditional in vivo methods fail to accurately classify a majority of the investigated compounds. While IBOA is correctly classified as a sensitizer by the LLNA, the method fails to identify the hazards posed by ACMO, HPMA, and SO60. Similarly, guinea pig methods fail to identify the hazard of NNDMA, while providing conflicting results for ACMO and HPMA. As such, the wearable industry may indeed benefit from implementing NAM-based testing strategies in order to preserve and protect human health. In light of the above reported results, it is relevant to mention that the current study did not include any expected non-sensitizers. This represents a deliberate choice, since the aim of the study was to evaluate the potential of the GARDskin DR method for quantitative potency assessments, which require positive test substances, as well as generating data from a set of test materials that are clinically relevant (i.e., with known cases of allergic contact dermatitis) among patient and end-users of medical devices. Nevertheless, this should not constitute a major limitation. Although the chemicals included in the study are relevant to the medical device and wearable industries, they should not be considered particularly unique in terms of their chemical reactivity profiles or other properties that may influence their sensitization potential when compared with chemicals included in previously published datasets on GARDskin and GARDskin DR. Hence, the performance values reported above can be considered in the context of previously published sensitivity and specificity values, summarized in the publications by, e.g., Lee et al. [28] and in the OECD TG 442E [14] for GARDskin.

Considering the results of quantitative potency assessments using GARDskin DR, it is clear that chemicals associated with the domain exhibit a wide range of predicted potencies, spanning approximately two orders of magnitude. Thus, implementing strategies that transition from pure hazard assessment to risk assessment with higher resolution may be relevant to nuance the results from pre-clinical testing. Here, it was noted that while curated reference data in widely used databases for skin sensitization research are missing, GARDskin DR seemingly captures and reflects clinical experience by classifying IBOA, SO60, and 23EPMA as relatively stronger sensitizers. While in vivo data are either lacking or largely unable to substantiate human potency thresholds, assessments using Defined Approaches (DA) for skin sensitization [29,31] provide another relevant source for comparison. DA potency predictions were recently published for a subset of the chemicals [59], relying on kDPRA [12] and KeratinoSens [13] data as predictors. In addition, SARA-ICE PoDs were generated as a third source of potency information, using the same NAM-derived inputs. Overall, the correlations between all models were very high, ranking IBOA and SO60 as the strongest skin sensitizers, followed by ACMO and then NNDMA. SARA-ICE was consistently the most conservative model, generating potency predictions 4 to 5 times lower than predictions from either the regression model or the GARDskin DR assay on this subset of chemicals. The largest differences observed between methods appeared to be driven less by the underlying NAM data than by differences in model specification and data interpretation. This was evident for the two defined approaches, which used identical input values yet generated potency predictions that differed more from each other than did the predictions from the GARDskin DR assay and the regression-based defined approach. Furthermore, it is noteworthy that in contrast to the DAs, which utilize a multitude of in vitro data sources, the single GARDskin DR assay produced highly comparable results. Indeed, such observations may be relevant as NAM-based approaches become implemented in non-regulatory screening settings by providing a perspective on resource efficiency.

Although wearables are typically not classified or regulated as medical devices (unless marketed with a medical purpose) and are therefore not subject to the stringent biological evaluations required by the ISO 10993 series of testing, they may still undergo extended pre-clinical safety testing to reduce risks to consumers. Similar to the testing of solid or semi-solid devices, the pre-clinical safety testing of wearables should ideally be performed on extracts from the materials, prepared in both polar and non-polar extraction vehicles as detailed in ISO 10993-12. The GARDskin Medical Device assay is currently the only OECD-validated assay that has demonstrated compatibility for testing in both polar and non-polar vehicles. Thus, to contextualize the above results from the GARDskin DR model, the chemical IBOA was further evaluated in a case study, in which it was spiked into pre-prepared proxy extracts of MED-2000 at the LLNA EC3 concentration. This procedure is identical to that proposed by ISO TS 11796, detailing the data-generating steps required to qualify OECD TG methods for standardized use according to ISO 10993-10. While the procedure does not evaluate an actual material containing the compound of interest *per se*, it adequately assesses a test method’s ability to detect the compound of interest in a complex formulation, as in routine and real-world scenarios. Here, the GARDskin MD method accurately identifies IBOA as a skin sensitizing hazard in both polar and non-polar extracts, demonstrating that the method is fit for purpose. Notably, it is worth emphasizing that the experimental design based on testing spiked extracts offers several practical and scientific advantages over testing commercially available devices. First of all, it is challenging to identify devices that are freely available on the market without prescription that can be used as positive controls. Secondly, the extraction profile from chemical characterization studies from such devices are often not publicly available, which would potentially make it challenging to evaluate the testing results. Finally, there may be variation between production batches or changes in the manufacturing process over time, potentially resulting in differences in the test materials when devices are tested from different batches or at different time points. Therefore, testing real devices for the purpose of validating alternative test methods is suboptimal. In contrast, generating proxy devices, by extracting a well-characterized reference material, and spiking the extract at fixed concentrations provides the added benefit of being able to generate reproducible experimental conditions over time, a critical feature enabling performance comparisons across different testing methods validated at different timepoints. In addition, since the concentrations of the spikes are pre-established, this also provides an opportunity to evaluate and compare the detection sensitivity between the historical animal tests and the new candidate method.

## 5. Conclusions

The current manuscript describes two novel applications of the OECD-validated GARDskin assay (OECD TG 442E), designed to address the specific testing requirements of wearables and medical devices. First, the GARDskin DR assay incorporates dose-response measurements to predict a PoD for quantitative risk assessments and to establish a safe threshold concentration. The testing of three acrylate monomers, two acrylamide monomers, and one reactive dye, all relevant to the wearable industry, demonstrated high concordance with available reference data, showing potential for applying a “safer-by-design approach” during the selection of materials or components for a final device. Furthermore, for the testing of medical devices, the ISO standard requires testing extracts from final devices, prepared in polar and non-polar extracts. A proof-of-concept study was conducted in which the chemical IBOA was spiked into extracts from MED-2000 to generate a proxy representative of a medical device. The resulting extracts were evaluated using the GARDskin Medical Device assay, demonstrating the potential to correctly identify the signal from IBOA in both the polar and non-polar vehicle. Thus, the herein presented adaptations of the GARD assays were shown to be functional tools to address the testing requirements for wearables and medical devices. These new tools have the potential to accelerate the transition from animal testing to the use of NAM-based strategies within the device and wearable sector.

## Figures and Tables

**Table 1:** Summary of reference data and GARDskin DR predictions.

Chemical ID	Summarized Reference Data ^1^		GARD Predictions		
	In Vivo	Human	cDV_0_ (µM)	PPV (µg/cm^2^)	CLP
ACMO	GPMT: POS, Buehler: NEG, LLNA: NEG	POS	84.8	3610	1B
HPMA	GPMT: POS/NEG, LLNA: NEG	POS	63.8	2770	1B
IBOA	LLNA: POS	POS	3.01	189	1A
NNDMA	GPMT: NEG	POS	465	13900	1B
SO60	LLNA: NEG	POS	4.35	354	1A
23EPMA	-	POS	10.9	468	1A

IBOA: isobornyl acrylate, ACMO: 4-acryloyl morpholine, NNDMA: N,N-dimethyl acrylamide, SO60: solvent orange 60, HPMA: hydroxypropyl methacrylate, EPMA: 2,3-epoxypropyl methacrylate. GPMT: Guinea Pig Maximization Test, LLNA: Local Lymph Node Assay. PPV: Predicted Potency Value, CLP: Classification, Labeling, and Packaging. ^1^: Reference details are available in Supplementary Material S1.

## Data Availability

The original contributions presented in this study are included in the article/Supplementary Materials. Further inquiries can be directed to the corresponding author.

## Supplementary Materials

The following supporting information can be downloaded at: https://www.mdpi.com/article/10.3390/toxics14100928/s1, S1: Review of available test chemical reference data [44,70,71,72,73,74].
