# Supplementary material for: Assessing the Skin Sensitizing Potential of Constituents and Leachables from Wearable Devices Using GARDskin and Associated Applications
Source: Toxics. 2026 Oct 20;14(10):928. doi: 10.3390/toxics14100928 (PMC13709857; doi:10.3390/toxics14100928)
Supplement: Supplementary file 1 [file toxics-14-00928-s001.zip › toxics-4553774-supplementary.pdf]

Supplementary Material

S1: Review of available test chemical reference data

N, N-dimethylacrylamide (NNDMA)

Negative in a GPMT when tested at 25% (Registration Dossier - ECHA). No LLNA data could be identified. Human data available from case reports indicate that NNDMA is a clinically relevant skin sensitizer, but likely in the lower potency range. For example, a case study by Mowitz and colleagues (https://doi.org/10.1111/cod.13243) reported that six of seven patients with contact dermatitis from the FreeStyle Libre glucose sensor reacted to both IBOA and NNDMA on patch testing, whereas one patient reacted only to NNDMA. Furthermore, in a follow-up study by Ulriksdotter and colleagues (DOI: 10.1111/cod.13649), 15 patients using the same sensor underwent patch testing with the Swedish baseline series and a medical device series. Positive reactions to IBOA and NNDMA were observed in 13 and 4 patients, respectively.

Hydroxypropyl methacrylate (HPMA)

The chemical structure of HPMA contains an α,β-unsaturated carbonyl group, which is a well-recognized alert for skin sensitization. Available data from animal studies, including two LLNA assays and several guinea pig studies, are (false) negative and indicate a lack of sensitization potential. However, the LLNA studies were tested only at a maximum concentration of 50%, so it is not possible to fully conclude a lack of weak sensitization potential (Registration Dossier - ECHA) . In addition, while most guinea pig tests were negative, a positive GPMT was reported in (https://pubmed.ncbi.nlm.nih.gov/6204493/). Moreover, the HPMA was evaluated in the Defined Approaches for skin sensitization and was classified as a skin sensitizer category 1B with high confidence (DASS Annex 2). Nevertheless, despite the negative animal data, HPMA is a well-documented skin sensitizer in humans. Case reports include workers exposed to curable inks containing the HPMA monomer (https://pubmed.ncbi.nlm.nih.gov/6204493/), as well as consumer nail product exposure (Reference: Screening for contact allergy to artificial nails). Based on the available data

presented above, the registration dossier suggests classification as a weak sensitizer with subcategorization 1B.

2,3-Epoxypropyl methacrylate

No animal data from studies conducted according to OECD Test Guidelines could be identified in the scientific literature. However, human data are available, indicating the skin sensitizing potential of 2,3-Epoxypropyl methacrylate. Three cases of ACD were reported from exposure to adhesive sealant during manufacturing; all were confirmed positive following patch testing with 2,3-Epoxypropyl methacrylate. Furthermore, there are reports of severe, sometimes bullous, ACD to 2,3-Epoxypropyl methacrylate and other methacrylates in exposed workers and consumers with positive patch tests, indicating a strong sensitization potential (doi: 10.1111/cod.12247). As per the ECHA registration dossier, 2,3-Epoxypropyl methacrylate is classified as a skin sensitizer, with the potency subcategorization 1A.

4-Acryloylmorpholine (ACMO)

Data from three guideline-compliant in vivo studies are available and demonstrated mixed results. First, a GPMT (OECD 406/EU method B.6) conducted at the highest non-irritant concentration produced evidence of skin sensitization in six of ten test animals, resulting in its classification as Cat 1 according to CLP criteria. Secondly, data were available from a Buehler test, which relies solely on topical exposure and is generally considered less sensitive than the GPMT, which includes intradermal injections and uses Freund's adjuvant. The test failed to induce any indications of skin sensitization in any of the ten exposed animals. Finally, data were available from an LLNA assay, where testing was performed up to 25% without evidence of sensitization. Nevertheless, as the guideline (OECD TG 429) recommends testing up to 100% or the highest achievable concentration, the study does not provide definitive evidence of non-sensitization. In summary, based on the data from the animal studies described above, the test substance was classified as a skin sensitizer Cat 1 as per the ECHA registration dossier (Registration Dossier - ECHA). In addition to the animal data, evidence from human cases

clearly indicates that ACMO is a skin sensitizer. The substance has been identified in a UV-curable adhesive used to attach protective films to smartwatches and has been associated with non-occupational allergic contact dermatitis (DOI: 10.1097/DER.0000000000000888). Furthermore, in an outbreak among workers handling smartphone screen protectors, seven individuals with suspected allergic contact dermatitis were tested with ACMO, and six showed positive reactions (DOI: 10.1111/cod.14079). Thus, based on available evidence, ACMO shall be considered a human skin sensitizer.

Solvent Orange 60 (SO60)

Animal data from an LLNA study conducted according to OECD TG 429 indicated a lack of skin sensitization potential. However, the highest evaluated concentration was 10%, and hence, a potential sensitizing effect if tested at a higher concentration cannot be ruled out (Registration Dossier - ECHA). Furthermore, several available case reports suggest that SO60 is a skin sensitizer in humans. First, a case study reports allergic contact dermatitis from exposure to SO60 present in earpieces of spectacle frames, which was confirmed by a positive patch test (DOI: 10.1111/j.1600-0536.1999.tb06126.x). These results were also further substantiated by a more recent study where patients with suspected ACD to eyewear were patch tested with SO60, and in five were confirmed positive (DOI:10.111/cod.13989). Furthermore, a retrospective review of 10 patients with suspected contact allergy to SO 60 from plastic spectacle frames found positive patch test responses in all cases. In one patient, serial dilution testing remained positive down to 1 ppm, indicating a strong sensitizing potential (DOI: 10.1111/cod.13022). Thus, based on the above, it is evident that SO60 is a skin sensitizer in humans.

Isobornyl acrylate (IBOA)

Data from a single LLNA study are availabe in the literature [1], which clearly indicates that IBOA is a skin sensitizer. All tested concentrations (5%,10% and 25%) were above the threshold value (SI=3), hence no exact EC3 value was determined in the study. Nevertheless, since the lowest tested concentration was positive (SI= 4.0), the EC3 is strictly lower than 5%, excluding a potency

subcategorization into any of the weaker potency groups (weak, very weak). Although it may be possible to further extrapolate the EC3 value based on the shape of the dose curve, it should be emphasized that the uncertainty of such an approximation would be high, especially considering that the study is not considered suitable for classification since the study was conducted using an expired batch of the test item, and results may be influenced by possible degradation product [2]. In addition to the positive animal data, human results from several case studies clearly indicate that IBOA is a skin sensitizer in humans. For example, IBOA is used in continuous glucose monitoring systems and insulin patch pumps, and several studies have identified this chemical as the causative agent for allergic reactions [3-4]. For example, 12 out of 13 patients with ACD attributable to the use of a flash glucose monitoring device showed positive reactions after patch testing with IBOA [3]. While available data conclusively identify IBOA as a skin sensitizer, less data are available for potency subcategorizations. However, a case study describes a patient that has first been exposed to a glucose monitor system and later also to an insulin patch pump. The patient developed skin reactions and was hence patch tested using the baseline series and plastic/glue series. All standard tests were negative, including the adhesives used for the devices. However, the patch test with IBOA gave a strong positive response (++). IBOA was not present in the adhesive, but it was identified in other parts of the insulin patch, at an amount corresponding to a surface dose/area of 0.53 µg/cm2 [5]. Although it is not clear if the patience was initially sensitized by this concentration, it deserves to mention that this is a very low threshold concentration, which would clearly assign IBOA to the more potent sensitization range. Furthermore, in another case study, four patients exposed to the same device, for which chemical analysis had confirmed the presence of IBOA (no quantitative data available), all showed positive patch test results when challenged with IBOA [6]. Overall, based on both animal and human data, IBOA may be considered a relatively potent sensitizer compared to the other chemicals described in the current dataset.

IBOA References:

70. ECHA Registration Dossier, available at: https://echa.europa.eu/lv/registration-dossier/-/registered-dossier/14494/7/5/2

71. German Federal Institute for Occupational Safety and Health (BAuA). (2019). CLH report for Exo-1,7,7-trimethylbicyclo[2.2.1]hept-2-yl acrylate (Isobornyl acrylate) (ECHA Doc. No. 10162/EA1FB91C-6702-4295-7152-76420C33BB75). European Chemicals Agency (ECHA)

72. Herman A., Aerts O., Baeck M., Bruze M., De Block C., Goossens A., Hamnerius N., Huygens S., Maiter D., Tennstedt D., Vandeleene B., and Mowitz M. (2017): Allergic contact dermatitis caused by isobornyl acrylate in Freestyle(R) Libre, a newly introduced glucose sensor. Contact Dermatitis 77 (6), 367-373. DOI: 10.1111/cod.12866

73. Corazza M., Scuderi V., Musmeci D., Foti C., Romita P., and Borghi A. (2018): Allergic contact dermatitis caused by isobornyl acrylate in a young diabetic patient using a continous glucose monitoring system (Freestyle Libre). Contact Dermatitis. DOI: 10.1111/cod.13075

74. Oppel E., Högg C., Summer B., Ruëff F., Reichl F.X., and Kamann S. (2018): Isobornyl acrylate contained in the insulin patch pump OmniPod as the cause of severe allergic contact dermatitis. Contact Dermatitis. DOI: 10.1111/cod.13017

75. Raison-Peyron N., Mowitz M., Bonardel N., Aerts O., and Bruze M. (2018): Allergic contact dermatitis caused by isobornyl acrylate in OmniPod, an innovative tubeless insulin pump. Contact Dermatitis 79 (2), 76-80. DOI: 10.1111/cod.12995
